# Case report: FGF4L1 retrogene insertion is lacking in the tall dachshund phenotype

**DOI:** 10.3389/fvets.2024.1522745

**Published:** 2025-01-03

**Authors:** Stacey Sullivan, Katarzyna Julia Szeremeta, Michelle Kutzler

**Affiliations:** ^1^College of Veterinary Medicine, Auburn University, Auburn, AL, United States; ^2^The International Working Teckel, Mannheim, Germany; ^3^Department of Animal and Rangeland Sciences, Oregon State University, Corvallis, OR, United States

**Keywords:** case report, CDDY, CDPA, chondrodystrophy, chondrodysplasia, FGF4L1, FGF4L2, limb-length

## Abstract

Two retrogene insertions, FGF4L1 (formerly 18-FGF4, colloquially CDPA) and FGF4L2 (formerly 12-FGF4, colloquially CDDY), have recently been discovered as determinants of short leg phenotype in dogs. This case study is comprised of a family of standard wirehaired dachshunds in which the dogs lacking the FGF4L1 gene exhibit a tall phenotype. The tall phenotype in the dachshunds of this report precludes the dog’s working function of den work. The data presented in this report provide information as to how FGF4L1 status could be used in making breeding decisions in dachshunds to maintain working ability without compromising animal health.

## Introduction

Canine morphology exhibits the widest variation of any mammal (1, 2) and in many breeds, morphology relates to working function. In dachshunds, a short leg phenotype was intentionally selected to enable the dog to work optimally in its historic hunting specialization: the pursuit of badger within dens, a function for which dachshunds are still used today (3). Dachshund literally translated from German means “badger dog.” With their shorter legs compared to terriers, dachshunds working underground can dig and pull prey more easily, illustrating that their body morphology serves a functional purpose (3).

Two retrogene insertions, FGF4L1 (formerly 18-FGF4, colloquially CDPA) and FGF4L2 (formerly 12-FGF4, colloquially CDDY), are associated with a short leg phenotype in most dog breeds (4, 5). The insertions are reported to behave in a dominant manner with a gene dose effect, meaning that one copy of either gene is sufficient to shorten limb length in dogs but that two copies have a more pronounced effect (5). FGF4L1 has a greater impact on limb length than does FGF4L2, with two copies of FGF4L1 reducing antebrachial length by 25%, while two copies of FGF4L2 reduces antebrachial length by only 10% (5, 6). It has been reported that some dog breeds such as beagles exhibit short leg phenotype due to FGF4L2 only, while other dog breeds such as dachshunds exhibit a shorter leg phenotype due to both FGF4L1 and FGF4L2 (5). A few dog breeds such as miniature pinschers exhibit short leg phenotype without either retrogene insertion. In addition to affecting limb length, FGF4L1 is associated with carpal valgus in dogs (5, 7) and FGF4L2 is associated with early disk degeneration (6, 8–10). In 697 dachshunds, allele frequency for FGF4L1 and FGF4L2 was reported to be 0.98 and 0.95, respectively, meaning that 96% of the dachshunds tested had 2 copies of FGF4L1 and 90% had 2 copies of FGF4L2 (11). Thus, most dachshunds are homozygous for both FGF4 retrogene insertions.

**Figure 1:**
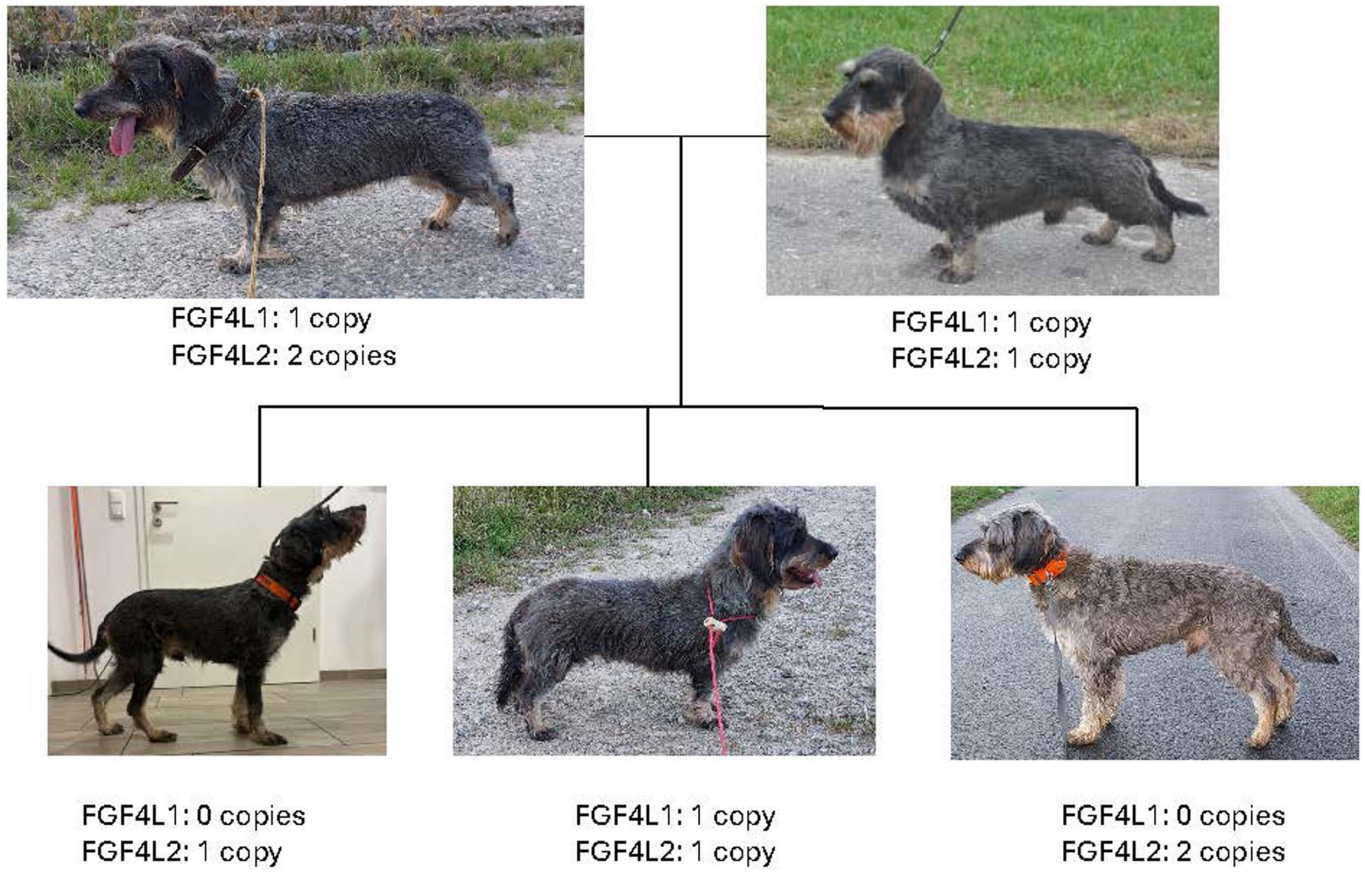
Family tree of the dachshunds including genotypes.

We report here a case of an FGF4L1 zero-copy standard dachshund with a tall phenotype (48 cm height at withers) that falls outside the upper limit (40 cm) (12) suitable for den work in earth dog breeds. We also report an FGF4L1 zero-copy sibling with a similar tall phenotype, and FGF4L1 one-copy sire, dam, and littermate with the typical dachshund short phenotype. Our findings suggest that at least one FGF4L1 copy is necessary in dachshunds to maintain a phenotype capable of the dog’s below-ground den hunting specialization (Figure 1).

### Timeline

**June 15, 2024**

Tall dachshund identified in response to social media request.

**August 6, 2024**

Height/weight data, and genotype (FGF4L1 and FGF4L2) obtained from case dog.

**August 10, 2024**

Genotype (FGF4L1 and FGF4L2) data obtained from sire, dam and female littermate of case dog.

**October 23, 2024**

Genotype (FGF4L1 and FGF4L2) obtained from male littermate of case dog.

### Narrative

A 5-year-old male intact standard wirehaired dachshund registered with the Deutscher Teckelklub was identified by one of the authors (KS) in response to a social media request for photographs of dachshunds of known FGF4 genotype for a book chapter on body morphology in working dachshunds. The dog was observed to lack the breed-typical short leg phenotype and was not immediately recognizable as a dachshund but was otherwise healthy. Height at withers was 48 cm, and weight was 17.5 kg (both approximately twice what is typical for a standard dachshund). Review of genotype data revealed the dog to have 0 copies of FGF4L1 and 1 copy of FGF4L2. The breeder of the dog was interviewed. The breeder had maintained ownership of the sire, dam, and a female littermate of the tall dachshund. It was reported that the sire and dam had been paired twice, and in each litter, multiple puppies of typical short leg phenotype were produced, along with one or two puppies of tall phenotype. The owner of a second tall dachshund, a male littermate to the case dog, was contacted. Height at withers for this second tall dachshund was 41 cm and weight was 11.7 kg. Each dachshund owner provided photographs of their dog(s) and FGF4L1 and FGF4L2 genotype data. Genotype and phenotype data are summarized in Table 1. Each phenotype group (short and tall) contained dogs with 1 or 2 FGF4L2 copies. However, no tall dachshund had the FGF4L1 insertion, while all of the short dachshunds had the FGF4L1 insertion. All of the dogs described in this report were healthy and no diagnostic tests or therapeutics were indicated.

**Table 1:** Phenotype and FGF4L1 & FGF4L2 Genotype for the Dachshund Family.

Dog	Phenotype (height)	Genotype	Genotype
		FGF4L1 Copies	FGF4L2 Copies
Case	Tall (48 cm)	0	1
Male littermate	Tall (41 cm)	0	2
Sire	Short	1	1
Dam	Short	1	2
Female littermate	Short	1	1

## Discussion

To our knowledge, this is the first report describing the phenotype of dachshunds lacking a copy of the FGF4L1 gene. The dachshunds presented in this report illustrate that a genotype lacking FGF4L1 may result in a tall phenotype unsuitable for den work. It is not known if this tall phenotype could result in musculoskeletal disorders later in life. Interestingly, the smaller of the two reported tall dachshunds carried two FGF4L2 copies and the taller individual carried only one FGF4L2 copy. This is consistent with the previously reported gene dose effect for limb length for these insertions (5), which predicts that a dog with four doses of these retrogene insertions (two copies of both FGF4L1 and FGF4L2, the typical dachshund pattern) would be shorter than a dog with two doses (zero FGF4L1 + two FGF4L2, the typical beagle pattern). Of the dachshunds in this report, one had the typical beagle pattern (zero FGF4L1 + two FGF4L2) and was of similar height (41 cm) to a 15-inch (38 cm) beagle.

It has also been reported that FGF4L1 has a more profound effect on limb length than FGF4L2 (5). The morphology of the dachshunds in our report supports that claim. The dachshunds with two doses of these retrogene insertions in the pattern one FGF4L1 + one FGF4L2 were short, but the dachshund with two doses in the pattern zero FGF4L1 + two FGF4L2 was tall. It is noteworthy that the short dachshund with three doses of these retrogene insertions in the pattern one FGF4L1 + two FGF4L2 was not readily distinguishable from the other short dachshunds. Thus, the dogs of our report suggest that FGF4L1 in at least one copy is necessary to the breed-defining short leg phenotype of dachshunds. Additionally, these dogs suggest that, provided FGF4L1 is present, FGF4L2 copy number is a less important determinant of limb length in dachshunds and may even be unnecessary to produce a short leg phenotype. In contrast, FGF4L2 might be necessary for breed-defining short leg phenotype in breeds lacking FGF4L1 such as the beagle.

Although the zero-copy FGF4L1 genotype in dachshunds is rare, the description of the dogs in this report is particularly timely, given the fact that in October 2024, the German government banned the exhibition of dogs with one or two FGF4L2 copies, and possibly one or two FGF4L1 copies (13). In 2021, the Animal Welfare Dog Regulations (Tierschutz-Hunderverordung) (14) were revised in Germany to prohibit exhibition of dogs that are the product of so-called “torture breeding” (qualzucht), which is the breeding of dogs with any inherited defect. In October 2024, implementation guidelines for the law were made public. The guidelines, which are intended to clarify the expectations for implementation by state veterinarians, list both FGF4L1 and FGF4L2 as “torture breeding traits” (qualzuchtmerkmale). It is unclear whether the guidelines ban dogs with one or two copies of both insertions, or only the FGF4L2 insertion. Regardless, the guidelines amount to a ban of many short leg dogs, including most dogs of breeds (such as dachshunds) with a high FGF4L2 gene frequency. Banned dogs are excluded from breed shows, working trials or any activity that has a competitive character or attracts an audience. Some of these activities are necessary Deutscher Teckelklub prerequisites for breeding in dachshunds.

A short leg phenotype itself, however, is not a defect, as illustrated by its common occurrence in nature in various species of animals such as the badger, weasel, otter, skink, turtles, and most rodents, where it conveys an adaptive advantage in moving through burrows, climbing, and swimming, and by lowering the animal’s center of gravity (15, 16). Carpal valgus, although associated with FGF4L1 genotype, is uncommonly a clinical problem in dachshunds and is considered a fault according to the breed standard (17, 18). In 30 dachshunds, thoracic limb angular limb deformity was noted upon radiographic evaluation, but lameness, elbow incongruity, and osteoarthritis were uncommonly present (19), suggesting that thoracic limb shape may differ in dachshunds compared to other breeds without causing welfare concerns.

In contrast to FGF4L1 which is of questionable health significance in dachshunds, a genotype comprised of one or two FGF4L2 copies poses greater welfare concerns due to the association with intervertebral disk disease (IVDD) risk. However, the association between FGF4L2 and IVDD risk is a recent discovery (6) and the science on the subject is rapidly evolving. Within the last 2 years, evidence for a gene dose effect for FGF4L2-associated IVDD risk has been reported (10, 20). The gene dose evidence suggests stakeholders of breeds with high FGF4L2 allele frequency could adopt a strategy of initially breeding for FGF4L2 heterozygosity to reduce incidence of symptomatic disk disease and avoid genetic bottleneck, and then breed for zero copies once the frequency of the wild type allele increases. The dogs of our report suggest that lack of FGF4L2 may not preclude breed-defining short leg phenotype in dachshunds, provided FGF4L1 is present. The dachshund is a healthy breed other than FGF4L2-associated IVDD risk, with a longer life expectancy than most breeds of dogs, including mixed breeds and non-chondrodystrophic breeds (21–25). For this reason, it seems prudent to allow breed clubs time to adopt spinal health breeding schemes based on recent scientific advancements.

## Conclusion

The dachshunds described in this report demonstrate that lack of FGF4L1 retrogene insertions may result in a tall phenotype that renders the dogs unsuitable for den work, the hunting niche for which the breed was developed. The scientific literature does not support a dachshund breed ban based on FGF4L1 genotype, since evidence for significant adverse health consequence of one or two FGF4L1 copies is lacking.

## Data Availability

The original contributions presented in the study are included in the article/supplementary material, further inquiries can be directed to the corresponding author.
